# Novel Group of AChE Reactivators—Synthesis, In Vitro Reactivation and Molecular Docking Study

**DOI:** 10.3390/molecules23092291

**Published:** 2018-09-07

**Authors:** David Malinak, Eugenie Nepovimova, Daniel Jun, Kamil Musilek, Kamil Kuca

**Affiliations:** 1 Department of Chemistry, Faculty of Science, University of Hradec Kralove, Rokitanskeho 62, 500 03 Hradec Kralove, Czech Republic; david.malinak@uhk.cz (D.M.); eugenie.nepovimova@uhk.cz (E.N.); 2 University Hospital in Hradec Kralove, Sokolska 581, 500 05 Hradec Kralove, Czech Republic; daniel.jun@unob.cz; 3 Department of Toxicology and Military Pharmacy, Faculty of Military Health Sciences, University of Defence, Trebesska 1575, 500 01 Hradec Kralove, Czech Republic

**Keywords:** organophosphate, acetylcholinesterase, reactivation, oxime, in vitro, molecular docking

## Abstract

The acetylcholinesterase (AChE) reactivators (e.g., obidoxime, asoxime) became an essential part of organophosphorus (OP) poisoning treatment, together with atropine and diazepam. They are referred to as a causal treatment of OP poisoning, because they are able to split the OP moiety from AChE active site and thus renew its function. In this approach, fifteen novel AChE reactivators were determined. Their molecular design originated from former K-oxime compounds K048 and K074 with remaining oxime part of the molecule and modified part with heteroarenium moiety. The novel compounds were prepared, evaluated in vitro on human AChE (*Hss*AChE) inhibited by tabun, paraoxon, methylparaoxon or DFP and compared to commercial *Hss*AChE reactivators (pralidoxime, methoxime, trimedoxime, obidoxime, asoxime) or previously prepared compounds (K048, K074, K075, K203). Some of presented oxime reactivators showed promising ability to reactivate *Hss*AChE comparable or higher than the used standards. The molecular modelling study was performed with one compound that presented the ability to reactivate GA-inhibited *Hss*AChE. The SAR features concerning the heteroarenium part of the reactivator’s molecule are described.

## 1. Introduction

**Figure 1:**
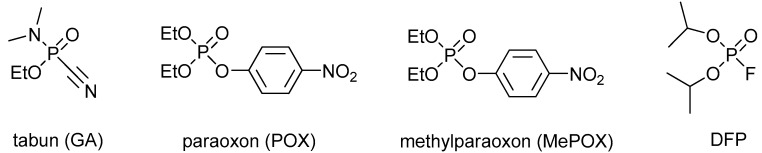
Selected organophosphorus compounds.

Organophosphorus agents (OP) are among the most toxic compounds developed by humans. They were originally discovered as pesticides (e.g., paraoxon, methylparaoxon; Figure 1), but they were further introduced as nerve agents (e.g., tabun, sarin, soman, VX; Figure 1), with highly lethal effects [1]. Some OPs are also utilized as industrial agents (e.g., flame retardants). Their mechanism of action is based on the irreversible inhibition of cholinesterases [2]. The toxic effect is caused by inhibition of acetylcholinesterase (AChE), while inhibited butyrylcholinesterase (BChE) takes part as an OP bioscavenger [3]. The inhibited AChE cannot fulfil its native function of degrading the neurotransmitter acetylcholine. Thus, acetylcholine is accumulated in the synapses, causing permanent stimulation of the cholinergic (muscarinic or nicotinic) receptors. For this reason, cholinergic symptoms (e.g., miosis, salivation, lacrimation, breath depression) occur that may worsen to cholinergic crisis or death from the malfunction of breathing muscles and the depression of the breathing center in the central nervous system [4].

**Figure 2:**
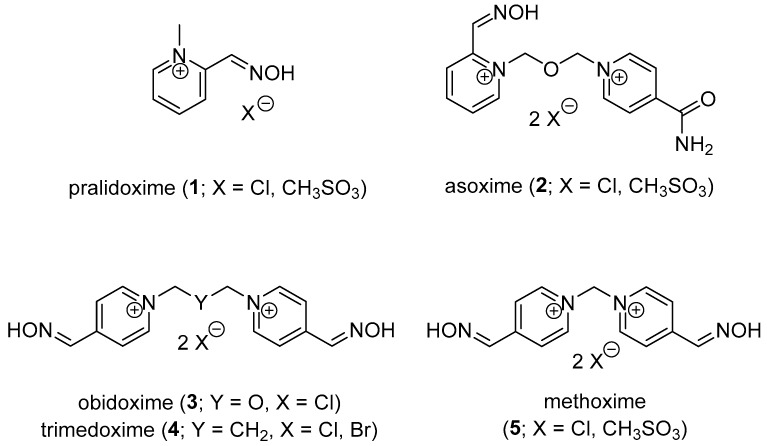
Commercially available AChE reactivators.

OP intoxication is usually managed using two treatment strategies. The first pre-treatment strategy for first responders (e.g., soldiers, medical staff) consists in the protection of the organism against OP attack. Pre-treatment can be performed using reversible AChE inhibitors (e.g., pyridostigmine chloride), stoichiometric scavengers (e.g., recombinant BChE) or catalytic bioscavengers (e.g., phosphotriesterase, paraoxonase) [5,6]. The second post-treatment strategy consists of managing already-OP-intoxicated organisms. The symptomatic post-treatment is focused on the amelioration of the OP intoxication symptoms [2]. Atropine (parasympatholytic drug) and diazepam (anticonvulsive drug), connected to supportive care, are usually used for this purpose [4]. The causal post-treatment is provided by oxime reactivators (e.g., pralidoxime, asoxime, obidoxime, trimedoxime, methoxime; **1**–**5**; Figure 2) via nucleophilic attack of the oximate towards the OP-AChE complex [7]. Such drugs are able to split the OP moiety from the AChE active site and restore its native function. The reactivation depends on many factors, e.g., OP dose, type of reactivator and its potency against certain OP, delay of reactivation treatment [8]. In fact, there is not a single reactivator available for a broad variety of OP compounds. The treatment is made even more complicated by the process of aging [9]. The OP moiety bound to the cholinesterase active site is further coordinated via loss of one ester moiety (*O*-dealkylation). Negatively charged oxygen appears, and the nucleophilic oxime reactivator is further ineffective. The aging process is well documented for nerve agents (over a time scale from minutes to hours), but some pesticides (e.g., methamidophos) may also be dealkylated in a similar manner [10].

Though the asoxime (**2**) was formerly found to be the most broad-spectrum oxime reactivator for nerve agent poisonings, it is not able to counteract, e.g., tabun or OP pesticide intoxication [11]. Thus, many scientific teams are trying to develop reactivators for nerve agent-inhibited AChE and/or OP pesticide poisoning. Recently, both quaternary and non-quaternary AChE reactivators have been considered for possible management of OP intoxication, including tabun exposure [12,13].

## 2. Design and Synthesis Mono-Oxime Bispyridinium Reactivators

**Figure 3:**
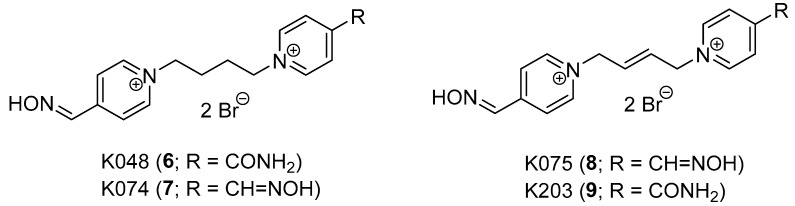
Promising formerly developed AChE reactivators.

The development of mono-oxime AChE reactivators with tetramethylene linker is described in this paper. The design of this series of compounds originated from the former series of oxime reactivators with (*E*)-but-2-en-1,4-diyl (**6**–**7**) and but-1,4-diyl (**8**–**9**) linkage, which showed some promising compounds for further study by in vitro and in vivo experiments (Figure 3) [14,15].

The nerve agent tabun (GA) remains the main OP of reactivation interest, followed by the selected OP pesticides. The previous structural findings showed that mono-oxime bispyridinium compounds (**6**, **9**) may have similar reactivation abilities against tabun-inhibited AChE as the known bis-oximes (**3**–**4**) [16,17]. Furthermore, the connection linkage at a length of 3–4 C-C bonds between two pyridinium moieties was considered to be optimal for GA reactivation [18]. For this reason, prop-1,3-diyl, but-1,4-diyl and (*E*)-but-2-en-1,4-diyl linkers were previously used [14,15]. The but-1,4-diyl linkage was the spacer that best fulfilled the found length criterion. Additionally, the bis-oxime compound with but-1,4-diyl linker (**7**) was previously found to be a potent reactivator of GA-inhibited AChE, although it resulted in increased toxicity during the in vivo studies [19,20].

**Figure 4:**
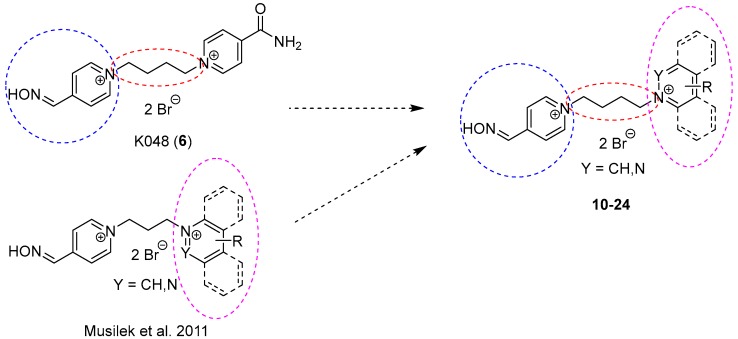
Design of bisquaternary mono-oxime reactivators with but-1,4-diyl linkage.

For the above-mentioned reasons, bisquaternary mono-oxime compounds with but-1,4-diyl linkage and varying second pyridinium ring or moiety on the second pyridinium ring were designed (**10**–**24**; Figure 4).

This second moiety was designed in the 4-position of the second pyridinium ring to allow the reactivator molecule to penetrate into the narrow AChE active site. The non-oxime moieties were chosen as representatives of hydrophilic (e.g., hydroxymethyl, methylcarbonyl, carboxyl, ethylcarboxyl, nitrile, amidoxime; **18**–**24**) and hydrophobic (e.g., methyl, tert-butyl, phenyl, benzyl; **14**–**17**) nature, or the whole pyridinium moiety was replaced by another heteroarenium ring (e.g., pyridazinium, quinolinium, isoquinolinium; **10**–**13**). The selected changes were designed to establish more possible interactions between reactivator and AChE, whether the π–cationic, π-π or hydrogen bonding interactions were the most common ones [21].

**Scheme 1:**
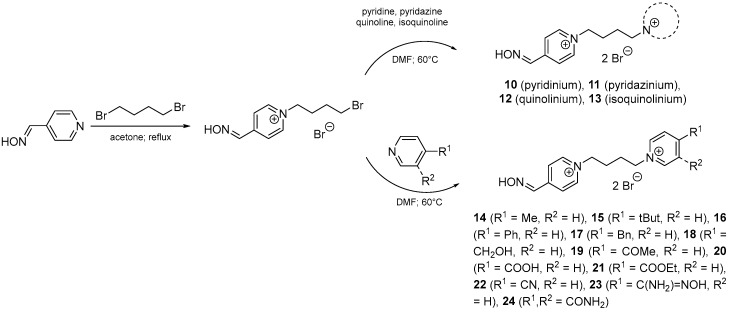
Synthesis of mono-oxime reactivators with tetramethylene linker.

The mono-oxime reactivators with tetramethylene linker were synthesized via a standard 2-step strategy (Scheme 1) [15]. The monoquaternary compound were prepared in 5 molar excess of the alkylating agent (1,4-dibromobutane). The monoquaternary compound was further separated from the side bisquaternary side product by crystallization from acetonitrile (MeCN), where the bisquaternary side product was almost insoluble. Secondly, the synthesis of mono-oxime reactivator was completed by the addition of appropriate heterocyclic agent (1.5 molar excess). The crystallization of MeCN was used again to give non-soluble bisquaternary reactivator (**10**–**24**) in a satisfactory purity and yield.

## 3. Results and Discussion

### 3.1. HssAChE Reactivation Results

The reactivation experiments were perofrmed on human recombinant AChE (*Hss*AChE) inhibited by tabun (GA), paraoxon (POX), methylparaoxon (MePOX) or diisopropylfluorophosphate (DFP) using the standard Ellman protocol [22]. GA was selected as the nerve agent of main interest. The chosen OP pesticides (POX, MePOX) and the mimic agent (DFP) were used, as they are distinct members of the OP family with remaining dimethyl-(MePOX), diethyl-(POX) or diisopropyl-(DFP) scaffolds after AChE inhibition. The reactivation results for all tested compounds are listed in Table 1.

The reactivation results for tabun-inhibited *Hss*AChE showed extensive differences, even in the group of commercial and known compounds [23]. Among these, pralidoxime (**1**), asoxime (**2**) and methoxime (**5**) showed poor reactivation ability [23]. On the other hand, obidoxime (**3**), trimedoxime (**4**) and compounds **6**–**9** displayed increased reactivation, with the best results being obtained for K203 (**9**; 48%) at 100 μM and K074 (**7**; 25%) at 10 μM concentration. Taken together with toxicity issues, some previously known AChE reactivators (e.g., **9**) have improved reactivation ability towards tabun both in vitro and in vivo [24]. The newly prepared compounds were prepared with the idea of improved tabun-inhibited *Hss*AChE reactivation. However, this objective was not fulfilled on the basis of the in vitro data for both tested concentrations, with the exception of compound **23**. Reactivator **23** (13%) showed better results than commercial compound **3** (8%) at 10 μM concentration, which tends to be attainable in blood after possible in vivo administration, but its activity was found to be worse than previously known compounds **4** (17%), **7**–**9** (25%, 18%, 21%) [25].

Furthermore, POX reactivation was tested. Not surprisingly, the commercial compounds **1**–**2** and **5** were found not to be effective in this case [26]. The other commercial compounds **3**–**4** presented increased reactivation of POX-inhibited *Hss*AChE at both determined concentrations (around 22% at 10 μM). The previously known compounds **6**–**9** showed lower reactivation ability up to 14% at human attainable concentration. The newly prepared compounds were found to be poor or average reactivators of POX-inhibited *Hss*AChE, with the exception of compounds **15** (59%) and **22** (43%) at 10 μM. These two compounds exceeded all commercially and previously known reactivators tested in this study against POX-induced inhibition.

The MePOX reactivation showed some similarities with POX reactivation. The commercial compounds **2** and **5** were found to be poor reactivators. However, surprisingly, the reactivation of compound **1** was found to be better at both concentrations (up to 30%) compared to POX reactivation (up to 11%). Nevertheless, commercial compounds **3**–**4** showed enhanced reactivation of MePOX-inhibited *Hss*AChE at human attainable concentration (45 and 60% at 10 μM) [15]. The previously known compounds **6**–**9** also as appeared to be promising MePOX reactivators, with a maximum of 41% at 10 μM for compound **9**. The newly prepared oximes showed decreased reactivation of MePOX-inhibited *Hss*AChE compared to the standards, with two compounds, **13** (17%) and **23** (21%), displayed some effect at 10 μM concentration.

The poor reactivation of DFP-inhibited *Hss*AChE was expected [15]. The previous experiments were confirmed by all standards **1**–**9**, where only compound **4** had some effective in vitro reactivation [27]. The newly prepared compounds also fulfilled these findings with minor or poor reactivation of DFP-inhibited *Hss*AChE.

When the reactivation data at various concentrations were taken into account, some compounds (e.g., **15**, **16**, **22**) were shown to be better reactivators at lower concentration (10 µM), compared to their reactivation data at higher concentration (100 µM). These reactivation results can be explained by mixed reactivation-inhibition behavior of newly prepared compounds at various concentrations [28]. For this purpose, the inhibitory effect towards *Hss*AChE of selected reactivators (**1**–**9**, **15**–**16** and **22**–**23**) was determined (Table 1). The commercial compounds **1**–**5** were shown to be poor *Hss*AChE inhibitors, with the best inhibitory ability exhibited by trimedoxime (**4**; 167 μM). Two previously known reactivators, **7** (29 μM) and **8** (80 μM), showed even better *Hss*AChE inhibition. Subsequently, compounds **4** and **7**–**8**, as the best AChE inhibitors among the standards, showed the above-mentioned mixed reactivation-inhibition effect for MePOX reactivation. Similarly, some newly prepared compounds (**15**–**16**, **22**) were found to be potent *Hss*AChE inhibitors. In accordance with our hypothesis, compound **16** (IC_50_ 6 µM) was shown to be a weaker reactivator of POX-inhibited *Hss*AChE at a concentration of 100 µM (7%) than at 10 µM (9%), while it greatly inhibited *Hss*AChE at the 100 µM concentration (screening concentration 100 µM >> IC_50_ 6 µM). At 10 µM, compound **16** was found to be a slightly better reactivator (9%), while its inhibition ability decreased ten-fold, and it was closer to its IC_50_ value (6 µM). Similarly, compound **15** (screening concentration 100 µM >> IC_50_ 24 µM) and compound **22** (screening concentration 100 µM >> IC_50_ 45 µM) confirmed the mixed reactivation-inhibition hypothesis during POX reactivation, while compound **23** (screening concentration 100 µM ≈ IC_50_ 90 µM) did not accurately fulfil this trend.

### 3.2. Molecular Modelling Results and SAR Discussion

The molecular modelling study was performed with oximes **3**, **9** and **23** to rationalize their interactions with GA-inhibited *Hss*AChE [29]. Oxime **3** was chosen as a known commercial standard that is able to reactivate GA-inhibited AChE [30]. Additionally, oxime **9** was selected from among the previously prepared compounds as one of the best reactivators of GA-inhibited *Hss*AChE [17]. Oxime **23** was selected as the best reactivator of GA-induced toxicity in vitro from among the newly prepared compounds.

The molecular modelling study was performed to better understand the in vitro results. The crystal structure of *Hss*AChE in complex with fasciculin 2 (pdb 1b41) was chosen as a crystal structure with good structural resolution (2.76 Å) [31]. The fasciculin molecule was deleted, while the molecule of GA was manually attached to the catalytic triad residue Ser203, and the whole molecule of GA-inhibited *Hss*AChE was minimized using Autodock Tools (ADT) [32]. The important aromatic residues within the *Hss*AChE active site were selected as flexible residues, including the GA-Ser203 moiety. The molecules of three reactivators were also minimized by ADT. The molecular modelling was performed by Autodock Vina [33].

**Figure 5:**
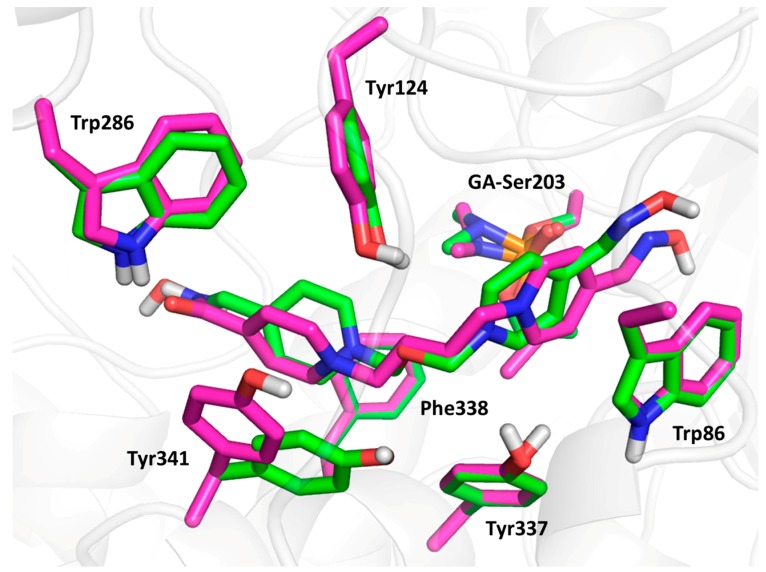
The molecular modelling results with selected flexible residues of compound **3** (in green) and compound **9** (in magenta).

**Figure 6:**
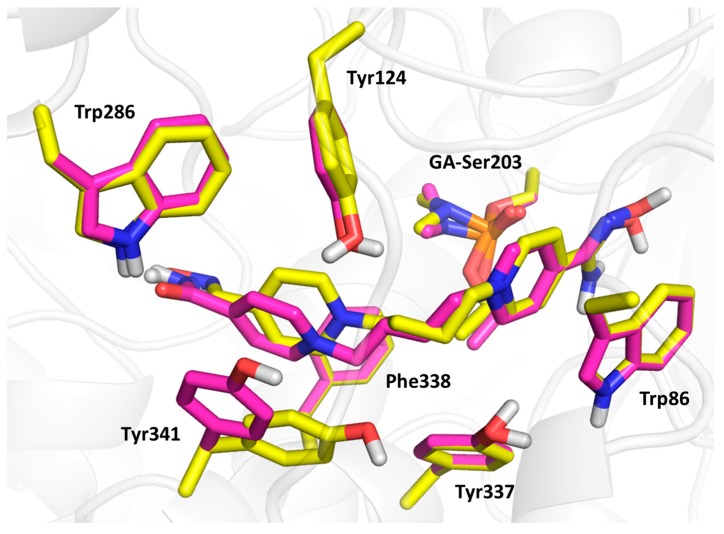
The molecular modelling results with selected flexible residues of compound **9** (in magenta) and compound **23** (in yellow).

Compound **3** (the lowest binding energy −5.7 kcal/mol) displayed typical interactions with AChE aromatic residues (Figure 5). The oxime-pyridinium ring was attached to Trp86 (3.6 Å), while the distance between oximate oxygen and GA phosphorus atom (6.8 Å) was found to be longer than previously observed results. The second pyridinium ring was stabilized via interactions with aromatic residues of the peripheral aromatic site (PAS), namely Phe338 (3.4 Å), Trp286 (4.2 Å), Tyr124 (4.0 Å) and Tyr341 (3.5 Å). The oxygen atom in the connecting linker seemed to be stabilized by interactions with phenolic moieties of Tyr124 (2.6 Å), Tyr337 (3.8 Å) and Tyr 341 (3.7 Å). Compound **9** (the lowest binding energy −5.5 kcal/mol) showed similar results to compound **3**, with some exceptions in terms of flexible residues (Figure 5 and Figure 6). In this case, the oxime pyridinium ring was stacked to Trp86 (3.6 Å) and the distance between the oxime and the GA phosphorus atom (5.1 Å) was better than previously published results. Not surprisingly, the non-oxime pyridinium ring was stabilized in PAS by Phe338 (3.6 Å), Trp286 (3.6 Å), Tyr124 (4.3 Å) and Tyr341 (3.6 Å), where the flexible residue Tyr341 was highly distorted compared to the same residue in the case of compound **3**. The double bond in the linker seemed to be stabilized by π-π interaction with Tyr337 (4.0 Å). The carbamoyl moiety displayed important hydrogen bonds with Trp286 (2.2 Å) and Phe295 (2.9 Å). Compound **23** (the lowest binding energy −6.4 kcal/mol) presented different binding when the amidoxime pyridinium ring was attached to Trp86 (3.6 Å), and amidoxime moiety was not located far from GA phosphorus (5.3 Å; Figure 6). The second ring bearing oxime moiety was stabilized in PAS by Phe338 (3.5 Å), Trp286 (3.7 Å), Tyr124 (3.6 Å) and Tyr341 (3.6 Å). The amidoxime moiety was further hydrogen bonded with Gly120 (3.3 Å) and Glu202 (2.1 Å).

Taken together, compound **9** showed more important interactions with GA-inhibited *Hss*AChE active sites compared to compounds **3** and **23** [29]. This might be a reason for its increased reactivation ability in the case of GA-induced in vitro inhibition. The lower reactivation efficacy of compound **3** might be explained by its different interactions within the GA-*Hss*AChE active site with inappropriate reactivator accommodation between Trp86 and PAS residues. Conversely, the lower in vitro reactivation efficacy of compound **23** might be related to the favorable structure with stabilized amidoxime moiety positioned close to GA-Ser203, while the spatial structure with oxime moiety positioned close to GA-Ser203 was found to be less favorable.

Concerning the SAR of oxime reactivators for GA or OP pesticide-inhibited *Hss*AChE, several important attributes should be considered. Firstly, the bisquaternary mono-oximes were already found to be satisfactory reactivators of GA- or pesticide-inhibited AChE compared to bis-oximes [34]. The 4-positioned oxime moiety on the pyridinium scaffold has been used and proposed many times as being optimal for reactivation due to its pKa [35]. Furthermore, the connecting linkage between heteroarenium rings in the length of 3–4 C-C atoms was highlighted in the case of GA- or pesticide-inhibited AChE [36]. In this case, the newly prepared compounds with 4-membered linkers showed results equivalent to previously prepared series with 3-membered linkers with analogous non-oxime moieties. Additionally, the hereroarenium part of the molecule without oxime moiety was found to be very important for proper reactivation effects in the case of various OPs. The second heteroarenium ring was determined to be an important moiety for interactions with aromatic amino acid residues in the PAS region. Its modification by pyridazinium, quinolinium or isoquinolinium moieties (**11**–**13**) did not lead to enhanced reactivation of GA or OP pesticide inhibited *Hss*AChE [15]. However, its modification by various moieties bound in the 4-position of pyridinium scaffold showed better reactivation compared to known and analogous bis-oxime **7**. Namely, some newly prepared compounds with hydrophobic (**15**-tertbutyl) or hydrophilic moieties (**22**-carbonitrile and **23**-amidoxime) were found to be promising reactivators of selected OP pesticide inhibited *Hss*AChE. Their increased reactivation ability might be explained by additional interactions with PAS amino acid residues, where tertbutyl (**15**) was proven to interact with aromatic residues, and carbonitrile (**22**) or amidoxime (**23**) are available for hydrogen bonding [37,38]. For the above-mentioned reasons, the second heteroarenium part of the molecule is the essential molecular fragment, and should be properly designed for enhanced reactivation of OP inhibited *Hss*AChE [34].

## 4. Experimental Section

### 4.1. Chemical Preparation

Solvents (acetone, DMF, MeCN) and reagents were purchased from Sigma-Aldrich (Prague, Czech Republic) and used without further purification. Reactions were monitored by TLC using DC-Alufolien Cellulose F (Merck, Darmstadt, Germany) and mobile phase BuOH-CH_3_COOH-H_2_O 5:1:2, detection by solution of Dragendorff reagent (solution containing 10 mL CH_3_COOH, 50 mL H_2_O and 5 mL of basic solution prepared by mixing of two fractions—fraction A: 850 mg Bi(NO_3_)_3_, 40 mL H_2_O, 10 mL CH_3_COOH; fraction B: 8 g KI, 20 mL H_2_O). Melting points were measured on micro heating stage PHMK 05 (VEB Kombinat Nagema, Radebeul, Germany) and were uncorrected.

NMR spectra were generally recorded at Varian Gemini 300 (^1^H 300 MHz, ^13^C 75 MHz, Varian, Palo Alto, CA, USA). In all cases, the chemical shift values for ^1^H spectra are reported in ppm (δ) relative to residual DMSO (δ 2.50) or D_2_O (δ 4.79), shift values for ^13^C spectra are reported in ppm (δ) relative to solvent peak dimethylsulfoxide—*d*_6_ δ 39.43. Signals are quoted as s (singlet), d (doublet), t (triplet) and m (multiplet).

The mass spectra (MS respectively MSn) were measured on a LCQ FLEET ion trap and evaluated using Xcalibur v 2.5.0 software (both Thermo Fisher Scientific, San Jose, CA, USA). The sample was dissolved in deionized water (Goro, s.r.o., Prague, Czech Republic), and injected continuously (8 µL/min) by Hamilton syringe into electrospray ion source. The parameters of electrospray were set up as follows: sheath gas flow rate 20 arbitrary units, aux gas flow rate 5 arbitrary units, sweep gas flow rate 0 arbitrary units, spray voltage 5 kV, capillary temperature 275 °C, capillary voltage 13 V, tube lens 100 V.

### 4.2. Preparation of Bisquaternary Salts

Initially the monoquaternary semi-product was prepared: a solution of the 4-hydroxyiminomethylpyridine (4.0 g, 32.8 mM) and 1,4-dibromobutane (19.6 mL, 163.8 mM) in acetone (30 mL) was stirred at reflux for 8 h [15]. The reaction mixture was cooled to room temperature and the crystalline crude product collected by filtration and washed with acetone (3 × 20 mL). The solid crude product was crystalized from MeCN (1 g of crude product per 100 mL of MeCN), where the boiling mixture was filtered under a reduced pressure. The filtrate was evaporated under a reduced pressure to produce the pure monoquaternary salt of 1-(4-bromobutyl)-4-hydroxyiminomethylpyridinium bromide (75%).

Secondly, the synthesis of the bisquaternary salt was completed. A solution of the monoquaternary salt (0.50 g, 1.5 mM) and selected heteroaromatic compound (3.0 mM) in DMF or MeCN (10 mL) was stirred at 70–80 °C for 8 h. The reaction mixture was cooled to room temperature and portioned with acetone (80 mL); the crystalline crude product was collected by filtration, washed with acetone (3 × 20 mL) and crystallized from boiling MeCN. The pure bisquaternary salt was obtained as the solid compound insoluble in boiling MeCN as the mixtures of cis/trans oxime isomers.

### 4.3. Prepared Monoquaternary Salt

*1-(4-Bromobutyl)-4-((hydroxyimino)methyl)pyridinium bromide.* M.p. 154–155 °C. Yield 91%. ^1^H-NMR (300 MHz, DMSO *d*_6_): δ (ppm) 12.82 (s, 1H, NOH), 9.08 (d, 2H, *J* = 6.5 Hz, H-2,6), 8.44 (s, 1H, -*CH*=NOH), 8.25 (d, 2H, *J* = 6.4 Hz, H-3,5), 4.64 (t, 2H, *J* = 7.2 Hz, N-*CH*_2_-), 3.57 (t, 2H, *J* = 6.6 Hz, *CH*_2_-Br), 2.08–2.01 (m, 2H, -*CH*_2_-), 1.86–1.80 (m, 2H, -*CH*_2_-). ^13^C-NMR (75 MHz, DMSO *d*_6_): δ (ppm) 148.32, 145.01, 144.88, 124.04, 59.25, 33.91, 29.29, 28.56. EA: calculated 35.53% C, 4.17% H, 8.29% N; found 35.58% C, 4.12% H, 8.25% N. ESI-MS: *m*/*z* 257.0 [M]^+^ (calculated [C_10_H_14_BrN_2_O]^+^ 257.03).

### 4.4. Prepared Bisquaternary Salts

*4-Hydroxyiminomethyl-1,1′-(but-1,4-diyl)-bispyridinium dibromide* (**10**) [39]. M.p. 243–244 °C. Yield 48%. ^1^H-NMR (300 MHz, DMSO *d*_6_): δ (ppm) 12.84 (s, 1H, NOH), 9.18 (d, 2H, *J* = 6.2 Hz, H-2,6), 9.12 (d, 2H, *J* = 6.2 Hz, H-2′,6′), 8.67-8.59 (m, 1H, H-4′), 8.46 (s, 1H, -*CH*=NOH), 8.25 (d, 2H, *J* = 6.2 Hz, H-3,5), 8.22–8.15 (m, 2H, H-3′,5′), 4.80–4.62 (m, 4H, N-*CH*_2_-), 1.97 (m, 4H, N-CH_2_-*CH*_2_-). ^13^C-NMR (75 MHz, DMSO *d*_6_): δ (ppm) 148.30, 145.53, 145.01, 144.98, 144.75, 128.05, 124.00, 59.63, 59.19, 27.06, 26.95. EA: calculated 43.19% C, 4.59% H, 10.07% N; found 43.29% C, 4.75% H, 10.08% N. ESI-MS: *m*/*z* 128.6 [M/2]^2+^ (calculated [C_15_H_19_N_3_O/2]^2+^ 128.58).

*4-Hydroxyiminomethyl-1,1′-(but-1,4-diyl)-1-pyridinium-1′-pyridazinium dibromide* (**11**). M.p. 222–224 °C. Yield 71%. ^1^H-NMR (300 MHz, DMSO *d*_6_): δ (ppm) 10.05 (d, 1H, *J* = 5.8 Hz, H-6′), 9.65 (d, 1H, *J* = 4.8 Hz, H-3′), 9.11 (d, 2H, *J* = 6.0 Hz, H-2,6), 8.80–8.73 (m, 1H, H-4′), 8.67–8.60 (m, 1H, H-5′), 8.45 (s, 1H, -*CH*=NOH), 8.26 (d, 2H, *J* = 6.0 Hz, H-3,5), 4.91 (t, 2H, *J* = 6.3 Hz, N’-*CH*_2_-), 4.68 (t, 2H, *J* = 6.3 Hz, N-*CH*_2_-), 2.10–1.96 (m, 4H, N-CH_2_-*CH*_2_-). ^13^C-NMR (75 MHz, DMSO *d*_6_): δ (ppm) 154.47, 150.12, 148.34, 145.03, 136.58, 136.01, 124.00, 63.44, 59.23, 26.98, 25.73. EA: calculated 40.21% C, 4.34% H, 13.40% N; found 40.09% C, 4.49% H, 13.27% N. ESI-MS: *m*/*z* 129.1 [M/2]^2+^ (calculated [C_14_H_18_N_4_O/2]^2+^ 129.08).

*4-Hydroxyiminomethyl-1,1′-(but-1,4-diyl)-1-pyridinium-1′-quinolinium dibromide* (**12**) [39]. M.p. 177–179 °C. Yield 21%. ^1^H-NMR (300 MHz, DMSO *d*_6_): δ (ppm) 9.68 (d, 1H, *J* = 5.8 Hz, H-2′), 9.34 (d, 1H, *J* = 8.4 Hz, H-8′), 9.12 (d, 2H, *J* = 6.2 Hz, H-2,6), 8.71 (d, 1H, *J* = 8.8 Hz, H-4′), 8.53 (d, 1H, *J* = 8.1 Hz, H-5′), 8.45 (s, 1H, -*CH*=NOH), 8.33–8.17 (m, 4H, H-3,3′,5,7′), 8.11–8.02 (m, 1H, H-6′), 5.17 (t, 2H, *J* = 7.0 Hz, N’-*CH*_2_-), 4.71 (t, 2H, *J* = 7.0 Hz, N-*CH*_2_-), 2.20–1.93 (m, 4H, N-CH_2_-*CH*_2_-). ^13^C-NMR (75 MHz, DMSO *d*_6_): δ (ppm) 149.71, 148.29, 147.41, 145.01, 137.33, 135.58, 130.70, 129.81, 129.66, 123.99, 122.16, 119.00, 59.33, 58.35, 27.36, 25.88. EA: calculated 48.85% C, 4.53% H, 8.99% N; found 48.41% C, 4.63% H, 9.06% N. ESI-MS: *m*/*z* 153.6 [M/2]^2+^ (calculated [C_19_H_21_N_3_O/2]^2+^ 153.59).

*4-Hydroxyiminomethyl-1,1′-(but-1,4-diyl)-1-pyridinium-1′-isoquinolinium dibromide* (**13**) [39]. M.p. 205–206 °C. Yield 26%. ^1^H-NMR (300 MHz, DMSO *d*_6_): δ (ppm) 10.26 (s, 1H, H-1′), 9.12 (d, 2H, *J* = 6.2 Hz, H-2,6), 8.87 (d, 1H, *J* = 6.7 Hz, H-3′), 8.64 (d, 1H, *J* = 6.7 Hz, H-4′), 8.51 (d, 1H, *J* = 8.2 Hz, H-8′), 8.44 (s, 1H, -*CH*=NOH), 8.38 (d, 1H, *J* = 8.2 Hz, H-5′), 8.32–8.21 (m, 3H, H-3,5,7′), 8.12–8.04 (m, 1H, 6′), 4.83 (t, 2H, *J* = 6.7 Hz, N-*CH*_2_-), 4.70 (t, 2H, *J* = 6.7 Hz, N’-*CH*_2_-), 2.18–1.94 (m, 4H, N-CH_2_-*CH*_2_-). ^13^C-NMR (75 MHz, DMSO *d*_6_): δ (ppm) 150.08, 148.30, 145.02, 136.92, 136.82, 134.85, 131.09, 130.33, 127.19, 127.13, 125.80, 124.99, 59.67, 59.29, 27.03, 26.81. EA: calculated 48.85% C, 4.53% H, 8.99% N; found 48.53% C, 4.75% H, 8.78% N. ESI-MS: *m*/*z* 153.6 [M]^2+^ (calculated [C_19_H_19_N_3_O]^2+^ 153.59).

*4-Hydroxyiminomethyl-4′-methyl-1,1′-(but-1,4-diyl)-bispyridinium dibromide* (**14**). M.p. 190–192 °C. Yield 47%. ^1^H-NMR (300 MHz, D_2_O): δ (ppm) 8.83 (d, 2H, *J* = 6.2 Hz, H-2,6), 8.65 (d, 2H, *J* = 6.0 Hz, H-2′,6′), 8.39 (s, 1H, -*CH*=NOH), 8.21 (d, 2H, *J* = 6.0 Hz, H-3,5), 7.89 (d, 2H, *J* = 6.0 Hz, H-3′,5′), 4.70–4.58 (m, 4H, N-*CH*_2_-,N’-*CH*_2_-), 2.23 (s, 3H, -*CH*_3_), 2.15–2.08 (m, 4H, N-CH_2_-*CH*_2_-). ^13^C-NMR (75 MHz, D_2_O *d*_6_): δ (ppm) 161.06, 149.47, 146.87, 145.09, 143.67, 129.43, 125.60, 61.09, 60.50, 30.89. EA: calculated 44.57% C, 4.91% H, 9.75% N, found 44.13% C, 5.00% H, 9.61% N. ESI-MS: *m*/*z* 270.0 [M^2+^ − H] (calculated for [C_16_H_21_N_3_O^2+^ − H] 270.17).

*4-Hydroxyiminomethyl-4′-(1,1-dimethylethyl)-1,1′-(but-1,4-diyl)-bispyridinium dibromide* (**15**). M.p. 146–148 °C. Yield 75%. ^1^H-NMR (300 MHz, D_2_O): δ (ppm) 8.83 (d, 2H, *J* = 6.2 Hz, H-2,6), 8.70 (d, 2H, *J* = 6.2 Hz, H-2′,6′), 8.38 (s, 1H, -*CH*=NOH), 8.21 (d, 2H, *J* = 6.2 Hz, H-3,5), 8.08 (d, 2H, *J* = 6.2 Hz, H-3′,5′), 4.68–4.59 (m, 4H, N-*CH*_2_-,-N’-*CH*_2_-), 2.14–2.08 (m, 4H, N-CH_2_-*CH*_2_-), 1.40 (s, 9H, -*CH*_3_). ^13^C-NMR (75 MHz, D_2_O *d*_6_): δ (ppm) 159.71, 158.49, 157.67, 155.86, 154.35, 134.61, 134.25, 70.23, 70.06, 39.88, 36.84. EA: calculated 48.22% C, 5.75% H, 8.88% N, found 47.91% C, 6.13% H, 8.54% N. ESI-MS: *m*/*z* 156.5 [M^2+^/2] (calculated for [C_19_H_27_N_3_O^2+^/2] 156.61).

*4-Hydroxyiminomethyl-4′-phenyl-1,1′-(but-1,4-diyl)-bispyridinium dibromide* (**16**). M.p. 236–238 °C. Yield 45%. ^1^H-NMR (300 MHz, DMSO *d*_6_): δ (ppm) 9.19 (d, 2H, *J* = 6.2 Hz, H-2,6), 9.12 (d, 2H, *J* = 6.2 Hz, H-2′,6′), 8.56 (d, 2H, *J* = 6.2 Hz, H-3,5), 8.46 (s, 1H, -*CH*=NOH), 8.26 (d, 2H, *J* = 6.2 Hz, H-3′,5′), 8.14–8.07 (m, 2H, Ph), 7.69–7.62 (m, 3H, Ph), 4.79–4.61 (m, 4H, N-CH_2_-, N’-*CH*_2_-), 2.10–1.94 (m, 4H, N-CH_2_-*CH*_2_-). ^13^C-NMR (75 MHz, DMSO *d*_6_): δ (ppm) 154.61, 148.32, 145.03, 144.79, 133.43, 132.09, 129.63, 128.07, 124.45, 124.02, 59.27, 58.81, 27.01, 26.98. EA: calculated 51.14% C, 4.70% H, 8.52% N; found 50.92% C, 4.96% H, 8.08% N. ESI-MS: *m*/*z* 166.6 [M/2]^2+^ (calculated [C_21_H_23_N_3_O/2]^2+^ 166.59).

*4-Hydroxyiminomethyl-4′-phenylmethyl-1,1′-(but-1,4-diyl)-bispyridinium dibromide* (**17**). M.p. not measured (amorphous). Yield 11%. ^1^H-NMR (300 MHz, DMSO *d*_6_): δ (ppm) 9.37 (d, 2H, *J* = 6.0 Hz, H-2,6), 9.14 (d, 2H, *J* = 6.0 Hz, H-2′,6′), 8.46 (s, 1H, -*CH*=NOH), 8.39 (d, 2H, *J* = 6.0 Hz, H-3,5), 8.27 (d, 2H, *J* = 6.0 Hz, H-3′,5′), 7.90–7.77 (m, 3H, Ph), 7.68–7.60 (m, 2H, Ph), 4.86–4.77 (m, 2H, N-CH_2_-), 4.75–4.66 (m, 2H, N’-*CH*_2_-), 2.10–2.00 (m, 6H, Ph-*CH_2_*-,N-CH_2_-*CH*_2_-). ^13^C-NMR (75 MHz, DMSO *d*_6_): δ (ppm) 151.63, 148.34, 145.74, 145.09, 145.03, 134.82, 134.05, 130.29, 129.12, 129.04, 126.83, 124.02, 59.91, 59.24, 27.05, 26.96. EA: calculated 52.09% C, 4.97% H, 8.28% N; found 52.33% C, 4.79% H, 8.64% N. ESI-MS: *m*/*z* 346.0 [M^2+^ − H] (calculated [C_22_H_25_N_3_O^2+^ − H] 346.20).

*4-Hydroxyiminomethyl-4′-hydroxymethyl-1,1′-(but-1,4-diyl)-bispyridinium dibromide* (**18**). M.p. 162–164 °C. Yield 45%. ^1^H-NMR (300 MHz, DMSO *d*_6_): δ (ppm) 9.22–9.01 (m, 4H, H-2,2′,6,6′), 8.45 (s, 1H, -*CH*=NOH), 8.25 (d, 2H, *J* = 6.0 Hz, H-3′,5′), 8.05 (d, 2H, *J* = 5.9 Hz, H-3,5), 4.81 (s, 2H, -*CH*_2_-OH), 4.76–4.60 (m, 4H, N-CH_2_-,N’-*CH*_2_-), 2.05–1.89 (m, 2H, N-CH_2_-*CH*_2_-). ^13^C-NMR (75 MHz, DMSO *d*_6_): δ (ppm) 148.31, 145.03, 144.98, 144.04, 124.29, 124.01, 61.07, 59.24, 59.01, 26.99. EA: calculated 42.98% C, 4.73% H, 9.40% N, found 42.63% C, 4.98% H, 9.04% N. ESI-MS: *m*/*z* 286.0 [M^2+^ − H] (calculated for [C_16_H_21_N_3_O_2_^2+^ − H] 286.16).

*4-Hydroxyiminomethyl-4′-methylcarbonyl-1,1′-(but-1,4-diyl)-bispyridinium dibromide* (**19**). M.p. 217–219 °C. Yield 18%. ^1^H-NMR (300 MHz, D_2_O): δ (ppm) 9.11 (d, 2H, *J* = 6.0 Hz, H-2′,6′), 8.84 (d, 2H, *J* = 6.0 Hz, H-2,6), 8.49 (d, 2H, *J* = 6.2 Hz, H-3′,5′), 8.38 (s, 1H, -*CH*=NOH), 8.21 (d, 2H, *J* = 6.0 Hz, H-3,5), 4.69–4.64 (m, 4H, N-*CH*_2_-, N’-*CH*_2_-), 2.80 (s, 3H, -*CH*_3_), 2.20–2.15 (m, 4H, N-CH_2_-*CH*_2_-). ^13^C-NMR (75 MHz, D_2_O *d*_6_): δ (ppm) 198.29, 149.54, 149.40, 146.79, 146.51, 146.48, 145.04, 127.10, 125.53, 61.72, 60.93, 30.80, 27.87, 27.24. EA: calculated 44.47% C, 4.61% H, 9.15% N, found 44.08% C, 4.77% H, 9.15% N. ESI-MS: *m*/*z* 149.5 [M^2+^/2] (calculated for [C_17_H_21_N_3_O_2_^2+^/2] 149.58).

*4-Carboxy-4′-hydroxyiminomethyl-1,1′-(but-1,4-diyl)-bispyridinium dibromide* (**20**). M.p. decomp. 230 °C. Yield 51%. ^1^H-NMR (300 MHz, DMSO *d*_6_): δ (ppm) 9.33 (d, 2H, *J* = 6.2 Hz, H-2,6), 9.12 (d, 2H, *J* = 6.2 Hz, H-2′,6′), 8.48 (d, 2H, *J* = 6.0 Hz, H-3,5), 8.45 (s, 1H, -*CH*=NOH), 8.25 (d, 2H, *J* = 6.0 Hz, H-3′,5′), 4.88–4.62 (m, 4H, N-CH_2_-, N’-*CH*_2_-), 2.06–1.90 (m, 4H, N-CH_2_-*CH*_2_-). ^13^C-NMR (75 MHz, DMSO *d*_6_): δ (ppm) 163.53, 148.33, 146.15, 145.37, 145.02, 127.28, 124.00, 60.00, 59.19, 27.08, 26.89. EA: calculated 41.67% C, 4.15% H, 9.11% N; found 41.34% C, 4.40% H, 9.24% N. ESI-MS: *m*/*z* 150.6 [M/2]^2+^ (calculated [C_16_H_19_N_3_O_3_/2]^2+^ 150.57).

*4-Ethylcarboxy-4′-hydroxyiminomethyl-1,1′-(but-1,4-diyl)-bispyridinium dibromide* (**21**). M.p. 174–176 °C. Yield 8%. ^1^H-NMR (300 MHz, DMSO *d*_6_): δ (ppm) 9.36 (d, 2H, *J* = 6.0 Hz, H-2,6), 9.11 (d, 2H, *J* = 6.0 Hz, H-2′,6′), 8.53 (d, 2H, *J* = 6.0 Hz, H-3,5), 8.45 (s, 1H, -*CH*=NOH), 8.25 (d, 2H, *J* = 6.0 Hz, H-3′,5′), 4.85–4.76 (m, 2H, N-*CH*_2_-), 4.72–4.63 (m, 2H, N’-*CH*_2_-), 4.44 (q, 2H, *J*_12_ = *J*_22_ = 7.0 Hz, COO-*CH*_2_-), 2.05–1.92 (m, 4H, N-CH_2_-*CH*_2_-), 1.37 (t, 3H, *J* = 7.0 Hz, -*CH*_3_). ^13^C-NMR (75 MHz, DMSO *d*_6_): δ (ppm) 162.00, 148.31, 146.28, 144.99, 144.96, 143.80, 127.16, 123.98, 62.89, 60.11, 59.16, 27.06, 26.85, 13.81. EA: calculated 44.19% C, 4.74% H, 8.59% N, found 43.84% C, 5.19% H, 8.10% N. ESI-MS: *m*/*z* 164.5 [M^2+^/2] (calculated for [C_18_H_23_N_3_O_3_^2+^/2] 164.59).

*4-Carbonitril-4′-hydroxyiminomethyl-1,1′-(but-1,4-diyl)-bispyridinium dibromide* (**22**). M.p. 247–249 °C. Yield 21%. ^1^H-NMR (300 MHz, DMSO *d*_6_): δ (ppm) 9.50 (d, 2H, *J* = 6.2 Hz, H-2,6), 9.12 (d, 2H, *J* = 6.2 Hz, H-2′,6′), 8.76 (d, 2H, *J* = 6.2 Hz, H-3,5), 8.45 (s, 1H, -*CH*=NOH), 8.25 (d, 2H, *J* = 6.0 Hz, H-3′,5′), 4.89–4.59 (m, 4H, N-CH_2_-, N’-*CH*_2_-), 2.10–1.88 (m, 4H, N-CH_2_-*CH*_2_-). ^13^C-NMR (75 MHz, DMSO *d*_6_): δ (ppm) 148.32, 146.23, 145.00, 130.96, 126.79, 124.06, 114.76, 60.60, 59.11, 26.90, 26.78. EA: calculated 43.46% C, 4.10% H, 12.67% N; found 43.16% C, 4.26% H, 12.51% N. ESI-MS: *m*/*z* 141.1 [M^2+^/2] (calculated [C_16_H_18_N_4_O^2+^/2] 141.08).

*4-(1-Amino-1-hydroxyiminomethyl)-4′-hydroxyiminomethyl-1,1′-(but-1,4-diyl)-bispyridinium dibromide* (**23**). M.p. 258 °C. Yield 51%. ^1^H-NMR (300 MHz, DMSO *d*_6_): δ (ppm) 9.15–9.07 (m, 4H, H-2, 2′,6,6′), 8.45 (s, 1H, -*CH*=NOH), 8.33 (d, 2H, *J* = 6.2 Hz, H-3,5), 8.25 (d, 2H, *J* = 6.0 Hz, H-3′,5′), 6.46 (s, 2H, -*NH*_2_), 4.72–4.61 (m, 4H, N-CH_2_-, N’-*CH*_2_-), 2.02–1.91 (m, 4H, N-CH_2_-*CH*_2_-). ^13^C-NMR (75 MHz, DMSO *d*_6_): δ (ppm) 148.31, 147.77, 146.92, 145.03, 144.99, 144.55, 124.01, 122.71, 59.23, 59.09, 26.94, 26.88. EA: calculated 40.44% C, 4.45% H, 14.74% N; found 40.16% C, 4.56% H, 14.35% N. ESI-MS: *m*/*z* 157.6 [M^2+^/2] (calculated [C_16_H_21_N_5_O_2_^2+^/2] 157.59).

*3,4-Dicarbamoyl-4′-hydroxyiminomethyl-1,1′-(but-1,4-diyl)-bispyridinium dibromide* (**24**). M.p. decomposition at 191–193 °C. Yield 59%. ^1^H-NMR (300 MHz, DMSO *d*_6_): δ (ppm) 9.50–9.26 (m, 2H, H-2′,6′), 9.13 (d, 2H, *J* = 6.2 Hz, H-2,6), 8.51–8.33 (m, 3H, -*CH*=NOH, H-3,5), 8.31–8.03 (m, 5H, -CO*NH*_2_, H-5′), 4.86–4.61 (m, 4H, N-CH_2_-, N’-*CH*_2_-), 2.16–1.88 (m, 4H, N-CH_2_-*CH*_2_-). ^13^C-NMR (75 MHz, DMSO *d*_6_): δ (ppm) 165.71, 164.01, 150.72, 148.60, 146.41, 145.43, 144.67, 134.39, 126.33, 124.30, 60.17, 59.48, 27.22, 27.09. EA: calculated 40.58% C, 4.21% H, 13.92% N; found 40.34% C, 4.35% H, 13.46% N. ESI-MS: *m*/*z* 171.6 [M^2+^/2] (calculated [C_17_H_21_N_5_O_3_^2+^/2] 171.58).

### 4.5. In Vitro Reactivation Assay

The reactivation ability was measured on a multichannel spectrophotometer Sunrise (Tecan, Salzburg, Austria). The previously used Ellman’s procedure was slightly adapted [22]. Standard polystyrene microplates with 96 wells (Nunc, Roskilde, Denmark) were chosen as reaction cuvettes. Recombinant *Hss*AChE (Sigma-Aldrich) was used throughout experiments. Tabun (GA; *O*-ethyl-*N*,*N*-dimethylphosphoramidocyanidate) was obtained from the Military Facility in Brno. Pesticides paraoxon (POX), methylparaoxon (MePOX) and diisopropylfluorophosphate (DFP) were purchased from Sigma-Aldrich (Prague, Czech Republic). 50 mM phosphate buffer pH 7.4 was used throughout all experiments.

The activity of the enzyme was adjusted to 0.002 U/µL. The inhibited enzyme was prepared directly before the testing of the potential reactivator. Phosphate buffer containing 1 mg/mL albumin, and a solution of enzyme (15 µL) was incubated with 1 µM GA or 10 µM POX or 10 µM MePOX or 100 µM DFP (5 µL) at room temperature to achieve 95% inhibition. The time of inhibition was selected with respect to the inhibition half-life of each inhibitor (GA—40 min, POX and MePOX—1 h, DFP—1 h 20 min).

One well was filled by the following chemicals: inhibited enzyme solution (20 µL), phosphate buffer (60 µL), 0.4 mg/mL 5,5′-dithio-bis-(2-nitrobenzoic)-acid (DTBN; 20 µL). Cholinesterase was reactivated by the addition of 100 μM or 10 μM oxime-reactivator dissolved in the phosphate buffer. Enzyme activity was measured after 15 min incubation via addition of 1 mM acetylthiocholine chloride (20 µL, ATChCl). Oximolysis was determined similarly by displacing enzyme with the phosphate buffer containing 1 mg/mL albumine. The microplate was gently shaken by the incorporated robotic system just before measurement. Absorbance was measured against phosphate buffer at 412 nm.

The reactivation ability was calculated according to the following equation.

(1) $$(\%)=\frac{A_{r}-A_{ox}}{A_{0}-A_{i}}\times 100$$

*A_r_* indicates absorbance at 412 nm provided by cholinesterase reactivated by reactivator; *A_ox_*—absorbance provided by oximolysis; *A*_0_—absorbance provided by intact cholinesterase; *A_i_*—absorbance provided by inhibited cholinesterase.

All measurements were performed in triplicate and the reactivation data are expressed as average value ± standard deviation (SD).

### 4.6. In Vitro Inhibition Assay

The multichannel spectrophotometer Sunrise (Tecan, Salzburg, Austria) was used for all measurements of *Hss*AChE activity. The previously optimized Ellman’s procedure was slightly adapted in order to estimate inhibitory properties *Hss*AChE reactivators [15]. 96-well photometric microplates made from polystyrene (Nunc, Roskilde, Denmark) were used for measuring purposes. Recombinant *Hss*AChE (Aldrich; commercially purified by affinity chromatography) was suspended in phosphate buffer (pH 7.4) to a final activity of 0.002 U/µL. Cholinesterase (5 µL), a freshly mixed solution of 0.4 mg/mL 5,5′-dithio-bis(2-nitrobenzoic) acid (40 µL), 1 mM acetylthiocholine chloride in phosphate buffer (20 µL) and the appropriate concentration of inhibitor (1 mM–0.1 nM; 5 µL) were injected per well. Absorbance was measured at 412 nm after 5 min incubation by automatic shaking of the microplate.

Percentage of inhibition (I) was calculated from the measured data as follows:

(2) $$I=1-\frac{\Delta A_{i}}{\Delta A_{0}}$$

Δ*A_i_* indicates absorbance change provided by cholinesterase exposed to anticholinergic compound. Δ*A*_0_ indicates absorbance change caused by intact cholinesterase, where phosphate buffer was applied in the same way as the anticholinergic compound.

IC_50_ was determined using Origin 6.1 (OriginLab Corporation, Northampton, MA, USA). Percentage of inhibition was calculated by Hill plot (*n* = 1). The other plot variants (between −2 and +2) were not optimal and the coefficient of determination was lower compared to chosen method. Subsequently, IC_50_ was computed.

### 4.7. Molecular Docking Study

The structure of *Hss*AChE was prepared from the crystal structure (pdb code b1b41) using Autodock Tools 1.5.6 (ADT) [31,32]. The fasciculine molecule was deleted and the molecule of tabun was manually added. The GA-inhibited *Hss*AChE was minimized and prepared for calculation using ADT. The 3D affinity grid box was designed to include the full active and peripheral site of *Hss*AChE. The number of grid points on the *x*-, *y*- and *z*-axes was 55, 55 and 55, with grid points separated by 0.400 Å. The important *Hss*AChE aromatic residues (His447, Phe295, Phe297, Phe338, Trp86, Trp236, Trp286, Tyr72, Tyr124, Tyr337 and Tyr341) and GA-inhibited Ser203 were set up as flexible residues. The molecular model of ligand was built using ChemDraw 11.1, minimized with UCSF Chimera 1.3 (Amber Force field) in charged form and prepared by ADT [40]. The calculation was done using AutoDock Vina 1.1.2 [33]. The pdb files were obtained with iBabel 2.6. The visualization of enzyme-ligand interactions (Figure 5 and Figure 6) was prepared using Pymol 1.1 [41].

## 5. Conclusions

A new series of mono-oxime *Hss*AChE reactivators with tetramethylene linkage was designed as analogues of previously prepared compounds K048 (**6**) and K074 (**7**). Fifteen newly prepared compounds were synthesized and determined on the in vitro model of GA, POX, MePOX and DFP-inhibited *Hss*AChE. The reactivation ability of the novel compounds was compared to the commercial standards and selected promising previously known compounds. Some novel compounds presented enhanced ability to reactivate OP-inhibited *Hss*AChE, while at least two of them (**15**, **22**) were able to exceed the reactivation potency of the already known compounds for POX-inhibited *Hss*AChE. One compound (**23**) presented promising reactivation of GA-inhibited *Hss*AChE. This reactivator was selected for a further molecular modelling experiment that confirmed the proposed and important interactions with PAS of *Hss*AChE.

## Figures and Tables

**Table 1:** Reactivation results of commercial, formerly published and newly prepared AChE reactivators.

	Non-Reactivating Moiety	GA-*Hss*AChE Reactivation (% ± SD)		POX-*Hss*AChE Reactivation (% ± SD)		MePOX-*Hss*AChE Reactivation (% ± SD)		DFP-*Hss*AChE Reactivation (% ± SD)		*Hss*AChE IC_50_ ± SD
Compound		100 μM	10 μM	100 μM	10 μM	100 μM	10 μM	100 μM	10 μM	µM
**1** (2-PAM)	-	3.3 ± 0.5	2.4 ± 0.2	10.7 ± 0.3	2.1 ± 0.1	30.2 ± 0.3	22.4 ± 0.7	1.3 ± 0.6	0.1 ± 0.4	878 ± 171
**2** (HI-6)	CONH_2_	0.9 ± 0.6	0.8 ± 0.3	6.2 ± 0.6	1.7 ± 0.1	13.6 ± 0.2	17.9 ± 0.4	0.7 ± 0.1	1.4 ± 0.1	203 ± 39
**3** (obidoxime)	CH=NOH	15.1 ± 0.9	7.9 ± 0.5	59.7 ± 1.0	22.4 ± 0.4	61.7 ± 0.3	45.3 ± 0.9	7.6 ± 0.7	3.3 ± 0.4	577 ± 113
**4** (trimedoxime)	CH=NOH	31.5 ± 1.2	16.9 ± 0.2	44.3 ± 0.6	22.5 ± 1.3	51.4 ± 0.9	59.5 ± 0.7	10.0 ± 0.5	2.7 ± 0.3	167 ± 33
**5** (methoxime)	CH=NOH	2.9 ± 0.01	2.1 ± 0.5	16.1 ± 0.5	1.8 ± 0.3	14.2 ± 0.1	14.3 ± 0.2	2.4 ± 0.1	0.6 ± 0.3	2010 ± 391
**6** (K048)	CONH_2_	27.4 ± 1.1	10.0 ± 0.6	25.7 ± 0.7	12.5 ± 0.2	54.4 ± 0.9	29.1 ± 0.4	3.8 ± 0.1	1.4 ± 0.1	349 ± 68
**7** (K074)	CH=NOH	32.1 ± 0.6	24.6 ± 0.2	31.1 ± 0.4	13.9 ± 0.8	17.9 ± 0.1	21.2 ± 0.4	1.4 ± 0.5	1.4 ± 0.2	29 ± 6
**8** (K075)	CH=NOH	19.5 ± 1.0	18.1 ± 0.2	28.5 ± 1.1	14.1 ± 0.3	19.2 ± 0.9	22.8 ± 0.2	1.1 ± 0.3	1.5 ±0.2	80 ± 16
**9** (K203)	CONH_2_	48.1 ± 1.5	21.2 ± 0.3	39.3 ± 0.4	13.1 ± 0.4	55.9 ± 0.5	41.1 ± 0.1	4.4 ± 0.1	2.1 ± 0.4	566 ± 110
**10**	pyridinium	3.9 ± 0.1	4.5 ± 0.3	16.6 ± 1.0	3.5 ± 0.3	14.3 ± 0.8	13.2 ± 0.1	2.4 ± 0.5	0.4 ± 0.3	-
**11**	pyridazinium	1.5 ± 0.5	4.1 ± 0.2	6.3 ± 0.8	3.0 ± 0.3	7.1 ± 0.7	11.8 ± 0.4	1.2 ± 0.7	1.0 ± 0.4	-
**12**	quinolinium	0.6 ± 0.4	3.1 ± 0.3	0.6 ± 0.5	2.0 ± 0.1	2.2 ± 0.5	8.6 ± 0.4	0.2 ± 1.0	0.8 ± 0.2	-
**13**	isoquinolinium	3.5 ± 0.7	6.0 ± 0.2	9.1 ± 0.3	8.7 ± 0.3	8.2 ± 0.8	16.9 ± 0.4	0.7 ± 0.2	1.1 ± 0.2	-
**14**	Me	3.4 ± 0.4	6.3 ± 0.3	24.5 ± 0.3	7.6 ± 0.6	9.5 ± 0.5	9.4 ± 0.2	1.1 ± 0.4	0.8 ± 0.3	-
**15**	tBut	1.7 ± 0.4	3.9 ± 0.3	18.7 ± 0.2	58.7 ± 4.0	4.0 ± 0.5	7.6 ± 0.5	1.7 ± 0.5	0.8 ± 0.3	24 ± 6
**16**	Ph	0.7 ± 0.4	3.5 ± 0.3	6.9 ± 0.4	8.9 ± 0.5	1.7 ± 0.3	6.2 ± 0.4	0.7 ± 0.2	1.0 ± 0.3	6 ± 1
**17**	Bn	0.3 ± 0.2	1.9 ± 0.6	1.2 ± 0.1	4.7 ± 0.3	0.0 ± 0.1	3.8 ± 0.4	0.0 ± 0.5	0.8 ± 0.2	-
**18**	CH_2_OH	2.6 ± 0.3	4.0 ± 0.3	20.6 ± 0.1	8.0 ± 0.1	6.7 ± 0.3	8.1 ± 0.3	3.8 ± 1.0	1.4 ± 0.4	-
**19**	COMe	1.7 ± 0.3	3.1 ± 0.1	11.9 ± 1.0	3.9 ± 0.3	5.2 ± 0.4	7.5 ± 0.1	1.5 ± 0.3	1.0 ± 0.2	-
**20**	COOH	3.3 ± 0.5	6.2 ± 0.3	14.5 ± 0.6	4.2 ± 0.4	9.5 ± 0.3	8.8 ± 0.4	1.1 ± 0.5	0.4 ± 0.1	-
**21**	COOEt	1.7 ± 0.5	4.1 ± 0.2	7.1 ± 0.7	4.0 ± 0.6	3.8 ± 0.4	7.2 ± 0.2	0.8 ± 0.3	1.1 ± 0.2	-
**22**	CN	3.6 ± 0.4	5.2 ± 0.2	10.1 ± 0.8	42.6 ± 0.1	11.1 ± 0.1	13.8 ± 0.3	1.7 ± 0.5	0.4 ± 0.2	45 ± 9
**23**	C(NH_2_)=NOH	12.8 ± 0.9	13.1 ± 0.4	19.1 ± 0.8	7.4 ± 0.3	20.2 ± 1.0	20.7 ± 0.4	3.1 ± 0.8	2.0 ± 0.2	90 ± 17
**24**	3,4-CONH_2_	1.8 ± 0.6	4.5 ± 0.2	2.0 ± 0.4	2.2 ± 0.8	4.2 ± 0.5	10.1 ± 0.2	0.7 ± 0.4	0.7 ± 1.0	-
